# Supplementary material for: Unconventional Hall effect induced by Berry curvature
Source: Natl Sci Rev. 2020 Jul 15;7(12):1879–85. doi: 10.1093/nsr/nwaa163 (PMC8288766; doi:10.1093/nsr/nwaa163)
Supplement: nwaa163_Supplemental_Files [file nwaa163_supplemental_files.zip › NSR-updated SI with highlights-0813.docx]

Supplementary Information for

**Unconventional Hall Effect induced by Berry Curvature**

Jun Ge^1,†^, Da Ma^1,†^, Yanzhao Liu^1^, Huichao Wang^1,2^, Yanan Li^1,3^, Jiawei Luo^1^, Tianchuang Luo^1^, Ying Xing^1,4^, Jiaqiang Yan^5,6^, David Mandrus^5,6^, Haiwen Liu^7^, X.C. Xie^1,8,9,10,*^, Jian Wang^1, 8,9,10, *^

*^1^ International Center for Quantum Materials, School of Physics, Peking University, Beijing 100871, China*

^2^ *Department of Applied Physics, The Hong Kong Polytechnic University, Kowloon, Hong Kong, China*

*^3^ Department of Physics, Pennsylvania State University, University Park, PA 16802*

*^4^ Department of Materials Science and Engineering, School of New Energy and Materials, China University of Petroleum, Beijing 102249, China*

*^5^ Department of Materials Science and Engineering, University of Tennessee, Knoxville, Tennessee 37996, USA*

*^6^* *Materials Science and Technology Division, Oak Ridge National Laboratory, Oak Ridge, Tennessee 37831, USA*

*^7^ Center for Advanced Quantum Studies, Department of Physics, Beijing Normal University, Beijing 100875, China*

*^8^ CAS Center for Excellence in Topological Quantum Computation, University of Chinese Academy of Sciences, Beijing 100190, China*

*^9^ Beijing Academy of Quantum Information Sciences, Beijing 100193, China*

*^10^ Collaborative Innovation Center of Quantum Matter, Beijing 100871, China*

*^9^ Beijing Academy of Quantum Information Sciences, Beijing 100193, China*

*^10^ Collaborative Innovation Center of Quantum Matter, Beijing 100871, China*

^†^These authors contributed equally to this work.

*email: jianwangphysics@pku.edu.cn (J.W.); xcxie@pku.edu.cn (X.C.X.)

**Supplementary notes on theoretical details**

1. **Semiclassical transport and theoretical fitting**

We investigate the electronic transport in ZrTe_5_ in the semiclassical regime. With Berry curvature $\boldsymbol{\Omega}_{\boldsymbol{k}}$ included, the equations of motion read [1-4]

$$\begin{aligned} \dot{\boldsymbol{r}}=\frac{1}{\hbar}\frac{\partial\epsilon}{\partial\boldsymbol{k}}-\dot{\boldsymbol{k}}\times\boldsymbol{\Omega}_{\boldsymbol{k}},\#(S1) \end{aligned}$$

$$\begin{aligned} \hbar\dot{\boldsymbol{k}}=-e\boldsymbol{E}-e\dot{\boldsymbol{r}}\times\boldsymbol{B,}\#\left( S2 \right) \end{aligned}$$

where $\dot{\boldsymbol{r}}$ is the group velocity of the wave packet, $\boldsymbol{k}$ is the momentum, $\boldsymbol{E}$ is the electric field, and $\boldsymbol{B}$ is the magnetic field. From the equations above, we can obtain

$$\begin{aligned} \dot{\boldsymbol{r}}=D\left( \boldsymbol{B},\boldsymbol{\Omega}_{\mathbf{k}} \right)[\boldsymbol{v}_{\boldsymbol{k}}+\frac{e}{\hbar}\boldsymbol{E}\times\boldsymbol{\Omega}_{\boldsymbol{k}}+\frac{e}{\hbar}\boldsymbol{(v}_{\boldsymbol{k}}\boldsymbol{\cdot}\boldsymbol{\Omega}_{\mathbf{k}}\boldsymbol{)B]},\#(S3) \end{aligned}$$

$$\begin{aligned} \hbar\dot{\boldsymbol{k}}=-D\left( \boldsymbol{B},\boldsymbol{\Omega}_{\mathbf{k}} \right)[e\boldsymbol{E}+e\boldsymbol{v}_{\boldsymbol{k}}\times\boldsymbol{B}+\frac{e^{2}}{\hbar}\boldsymbol{(E}\boldsymbol{\cdot}\boldsymbol{B)}\boldsymbol{\Omega}_{\mathbf{k}}\boldsymbol{],}\#\left( S4 \right) \end{aligned}$$

where $\boldsymbol{v}_{\boldsymbol{k}}=\frac{1}{\hbar}\frac{\partial\epsilon}{\partial\boldsymbol{k}}$ is the Fermi velocity, and $D\left( \boldsymbol{B},\boldsymbol{\Omega}_{\mathbf{k}} \right)=\left[ 1+\frac{e}{\hbar}\left( \boldsymbol{B}\boldsymbol{\cdot}\boldsymbol{\Omega}_{\mathbf{k}} \right) \right]^{-1}$ is the phase-space volume factor [1, 4]. In the presence of a magnetic field $\boldsymbol{B}$ and a Berry curvature $\boldsymbol{\Omega}_{\mathbf{k}}$, the phase space volume is not conserved, and the current density becomes [1, 4]

$$\begin{aligned} \boldsymbol{J}=-e\int\frac{d\boldsymbol{k}}{\left( 2\pi\right)^{3}}D^{-1}\dot{\boldsymbol{r}}f_{\boldsymbol{k}}\left( \boldsymbol{r} \right),\#\left( S5 \right) \end{aligned}$$

where $f_{\boldsymbol{k}}\left( \boldsymbol{r} \right)$is the distribution function of the electrons occupying the $\boldsymbol{k}$ state at the location $\boldsymbol{r}$ in real space, which is calculated from the Boltzmann equation with a relaxation time approximation,

$$\begin{aligned} \dot{\boldsymbol{k}}\cdot\nabla_{\boldsymbol{k}}f_{\boldsymbol{k}}\left( \boldsymbol{r} \right)=\frac{f_{\mathrm{eq}}\left( \boldsymbol{r} \right)-f_{\boldsymbol{k}}\left( \boldsymbol{r} \right)}{\tau\left( \boldsymbol{k} \right)}.\#\left( S6 \right) \end{aligned}$$

Here $\tau\left( \boldsymbol{k} \right)$ is the relaxation time, and $f_{\mathrm{eq}}\left( \boldsymbol{r} \right)$ is the distribution function at equilibrium. Substituting Eq. (S4) into Eq. (S6), we find

$$\begin{aligned} f_{\boldsymbol{k}}=f_{\mathrm{eq}}+\left[ eD\tau\boldsymbol{E}\cdot\boldsymbol{v}_{\boldsymbol{k}}+\frac{e^{2}}{\hbar}D\tau\left( \boldsymbol{E}\boldsymbol{\cdot}\boldsymbol{B} \right)\left( \boldsymbol{v}_{\boldsymbol{k}}\boldsymbol{\cdot}\boldsymbol{\Omega}_{\mathbf{k}} \right)\boldsymbol{+}\boldsymbol{v}_{\boldsymbol{k}}\boldsymbol{\cdot\Gamma} \right]\frac{\partial f_{\mathrm{eq}}}{\partial\epsilon},\#\left( S7 \right) \end{aligned}$$

where $\boldsymbol{\Gamma}$ is the higher-order correction term.

Next, we consider the Weyl semimetal with tilt term. For a pair of Weyl cones related by symmetry, the Hamiltonian around the Weyl point can be written as

$$\begin{aligned} H=\hbar v\boldsymbol{k}\cdot\boldsymbol{\sigma}+\hbar\boldsymbol{t}\cdot\boldsymbol{k}\#\left( S8 \right) \end{aligned}$$

for one cone, and

$$\begin{aligned} H=-\hbar v\boldsymbol{k}\cdot\boldsymbol{\sigma}-\hbar\boldsymbol{t}\cdot\boldsymbol{k}\#\left( S9 \right) \end{aligned}$$

for the other cone with the opposite chirality. Here $\boldsymbol{\sigma}$ is the vector of the Pauli matrices, and $\boldsymbol{t}$ is the tilt vector. Here we suppose that the tilt lies in the $xy$ plane, and thus the tilt term reads

$$\begin{aligned} \boldsymbol{t=}t\left( \cos\alpha,\sin\alpha,0 \right).\boldsymbol{\#}\left( S10 \right) \end{aligned}$$

Then, the Berry curvature of the tilt Weyl nodes in Eq. (S8) and Eq. (S9) can be obtained, which can be utilized to solve Eq. (S7) for the distribution function and give the final form for the conductivity as follows:

$$\sigma_{xx}=\frac{2e^{2}\tau}{\left( 2\pi\right)^{3}\hbar^{2}}\left[ -4\pi v\frac{t-v\tanh^{-1} \frac{t}{v}}{\hbar t^{3}}\mu^{2}\cos^{2} \alpha+\pi v^{2}\frac{2vt+2\left( t^{2}-v^{2} \right)\tanh^{-1} \frac{t}{v}}{\hbar t^{3}\left( v^{2}-t^{2} \right)}\mu^{2}\sin^{2} \alpha-4\pi etB\cos\alpha\cos\theta-\pi e\frac{-6t^{5}+10v^{2}t^{3}-6v^{4}t+6v\left( t^{2}-v^{2} \right)^{2}\tanh^{-1} \frac{t}{v}}{3t^{4}}B\cos^{2} \alpha\cos\left( \theta-\alpha\right)+\pi ev^{2}\frac{2t^{3}-3v^{2}t+3v\left( v^{2}-t^{2} \right)\tanh^{-1} \frac{t}{v}}{3t^{4}}B\sin^{2} \alpha\cos\left( \theta-\alpha\right)-2\pi e\frac{5v^{2}t^{3}-3v^{4}t+3v\left( t^{2}-v^{2} \right)^{2}\tanh^{-1} \frac{t}{v}}{3t^{4}}B\sin\alpha\cos\alpha\sin\left( \theta-\alpha\right)+\pi e^{2}\hbar\frac{t^{2}v+v^{3}}{\mu^{2}}B^{2}\cos^{2} \theta+\pi e^{2}\hbar\frac{7t^{2}v+v^{3}+\left( 4t^{2}v+2v^{3} \right)\cos^{2} \left( \theta-\alpha\right)}{15\mu^{2}}B^{2}\cos^{2} \alpha+\pi e^{2}\hbar\frac{v^{3}+2v^{3}\sin^{2} \left( \theta-\alpha\right)}{15\mu^{2}}B^{2}\sin^{2} \alpha-4\pi e^{2}\hbar\frac{t^{2}v+v^{3}}{15\mu^{2}}B^{2}\sin\alpha\cos\alpha\sin\left( \theta-\alpha\right)\cos\left( \theta-\alpha\right)-2\pi e^{2}\hbar\frac{13t^{2}v+5v^{3}}{15\mu^{2}}B^{2}\cos\alpha\cos\theta\cos\left( \theta-\alpha\right)+2\pi e^{2}\hbar\frac{t^{2}v+5v^{3}}{15\mu^{2}}B^{2}\sin\alpha\cos\theta\sin\left( \theta-\alpha\right) \right], \left( S11 \right)$$

$$\sigma_{yx}=\frac{2e^{2}\tau}{\left( 2\pi\right)^{3}\hbar^{2}}\left[ -4\pi v\frac{t-v\tanh^{-1} \frac{t}{v}}{\hbar t^{3}}\mu^{2}\sin\alpha\cos\alpha-\pi v^{2}\frac{2vt+2\left( t^{2}-v^{2} \right)\tanh^{-1} \frac{t}{v}}{\hbar t^{3}\left( v^{2}-t^{2} \right)}\mu^{2}\sin\alpha\cos\alpha-2\pi etB\sin\left( \alpha+\theta\right)-\pi e\frac{-6t^{5}+10v^{2}t^{3}-6v^{4}t+6v\left( t^{2}-v^{2} \right)^{2}\tanh^{-1} \frac{t}{v}}{3t^{4}}B\sin\alpha\cos\alpha\cos\left( \theta-\alpha\right)-\pi ev^{2}\frac{2t^{3}-3v^{2}t+3v\left( v^{2}-t^{2} \right)\tanh^{-1} \frac{t}{v}}{3t^{4}}B\sin\alpha\cos\alpha\cos\left( \theta-\alpha\right)+\pi e\frac{5v^{2}t^{3}-3v^{4}t+3v\left( t^{2}-v^{2} \right)^{2}\tanh^{-1} \frac{t}{v}}{3t^{4}}B\cos2\alpha\sin\left( \theta-\alpha\right)+\pi e^{2}\hbar\frac{t^{2}v+v^{3}}{\mu^{2}}B^{2}\sin\theta\cos\theta+\pi e^{2}\hbar\frac{7t^{2}v+v^{3}+\left( 4t^{2}v+2v^{3} \right)\cos^{2} \left( \theta-\alpha\right)}{15\hbar^{2}\mu^{2}}B^{2}\sin\alpha\cos\alpha-\pi e^{2}\hbar\frac{v^{3}+2v^{3}\sin^{2} \left( \theta-\alpha\right)}{15\hbar^{2}\mu^{2}}B^{2}\sin\alpha\cos\alpha-2\pi e^{2}\hbar\frac{t^{2}v+v^{3}}{15\hbar^{2}\mu^{2}}B^{2}\cos2\alpha\sin\left( \theta-\alpha\right)\cos\left( \theta-\alpha\right)-\pi e^{2}\hbar\frac{13t^{2}v+5v^{3}}{15\hbar^{2}\mu^{2}}B^{2}\sin\left( \theta+\alpha\right)\cos\left( \theta-\alpha\right)-\pi e^{2}\hbar\frac{t^{2}v+5v^{3}}{15\hbar^{2}\mu^{2}}B^{2}\cos\left( \theta+\alpha\right)\sin\left( \theta-\alpha\right) \right]. \left( S12 \right)$$

The Hall conductivity in Eq. (S12) can be utilized to fit the experimental data in Fig. 4f of the main text. The parameters in Fig. 4(f) are as follows: $v=4.6\times{10}^{5}$m/s, $t=4.0\times{10}^{5}$m/s, $\mu=8$meV, $\tau=0.4$ps, and $\alpha=-75.5^{\circ}$. Here, $v$ is Fermi velocity, t is the tilt of Weyl cones, $\mu$ is chemical potential, $\tau$ is relaxation time and$\alpha$ labels the angle between tilt vector and current. The relaxation time and the Fermi velocity are in agreement with other experiments.

1. **Low energy effective model of ZrTe_5_ in magnetic field**

Previous DFT calculation found that 3D ZrTe_5_ crystals are topological insulators [5]. Based on the DFT results, R. Y. Chen *et al.* constructed an effective model of ZrTe_5_ with symmetry analysis [6]. The effective model consists of four basis states as shown in Eq.(S13). Here $\tau_{z}=\pm1$ marks states of different orbitals, and $\sigma$’s are Pauli matrices in spin space. Linear expansion around $\Gamma$ point yields the following Dirac-like Hamiltonian, [6]

$$\begin{aligned} H_{0}=m\tau_{z}+\hbar\left( v_{x}k_{x}t_{x}\sigma_{z}+v_{y}k_{y}\tau_{y}+v_{z}k_{z}\tau_{x}\sigma_{x} \right),\#\left（ S13 \right） \end{aligned}$$

which describes the parent insulating state. Here $m$ is the Dirac mass, and $x, y, z$ axes above correspond to $a, c, b$ crystal axes, respectively.

In magnetic field, the Zeeman effect has to be considered, and there is an additional term in the Hamiltonian, [6]

$$H_{Zeeman}=-\frac{1}{2}\mu_{B}g\boldsymbol{\sigma}\cdot\boldsymbol{B},$$

where $\mu_{B}$ is Bohr magneton, and $g$ is the Landé g-factor. Further, we include some general quadratic dispersion around $\Gamma$ point,

$$H_{1}=C_{1}k_{x}^{2}+C_{2}k_{y}^{2}+C_{3}k_{z}^{2}.$$

For the complete Hamiltonian,

$$\begin{aligned} H=H_{0}+H_{1}+H_{Zeeman},\#\left（ S14 \right） \end{aligned}$$

the dispersion satisfies

$$\begin{aligned} E=C_{1}k_{x}^{2}+C_{2}k_{y}^{2}+C_{3}k_{z}^{2}\pm\sqrt{m^{2}+\hbar^{2}\left( \boldsymbol{v}\cdot\boldsymbol{k} \right)^{2}+\frac{\mu_{B}^{2}g^{2}B^{2}}{4}\pm\hbar\mu_{B}g\sqrt{\left( B_{z}v_{x}k_{x}+B_{x}v_{z}k_{z} \right)^{2}+B^{2}v_{y}^{2}k_{y}^{2}+B^{2}m^{2}},}\#\left（ S15 \right） \end{aligned}$$

where $\boldsymbol{v}=\left( v_{x}, v_{y}, v_{z} \right)$, and $\boldsymbol{k}=\left( k_{x}, k_{y}, k_{z} \right)$.

To incorporate with our experiment setup that the magnetic field lies in the $ac$-plane (the $xy$-plane here), we set $B_{z}=0$. When the magnetic field is larger than the critical value $B_{c}=\left| \frac{2m}{\mu_{B}g} \right|$, the Hamiltonian above describes a time-reversal-symmetry breaking Weyl semimetal, with a pair of Weyl points at $\left( 0, \pm\sqrt{\frac{\mu_{B}^{2}g^{2}B^{2}}{4}-m^{2}}, 0 \right)$. The quadratic term $C_{2}k_{y}^{2}$ in $H_{1}$ provides opposite additional velocities around the two Weyl points, which practically makes the two Weyl cones tilt along $y$-axis.

**Figures**


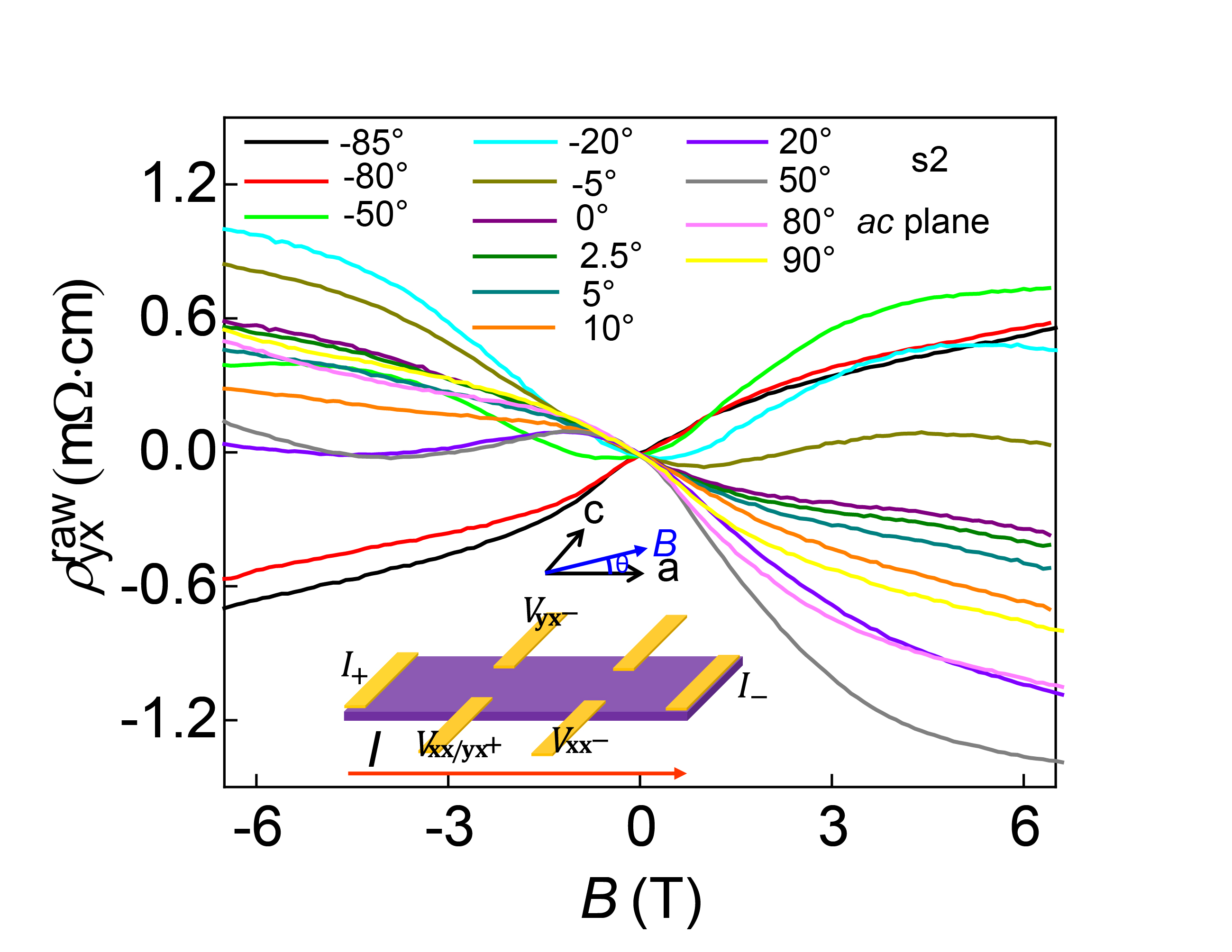


Fig. S1. Raw Hall data of ZrTe_5_ device s2 detected at selected angles in PPMS. Inset shows the schematic structure for the angular-dependent magnetotransport measurements in *ac* plane.


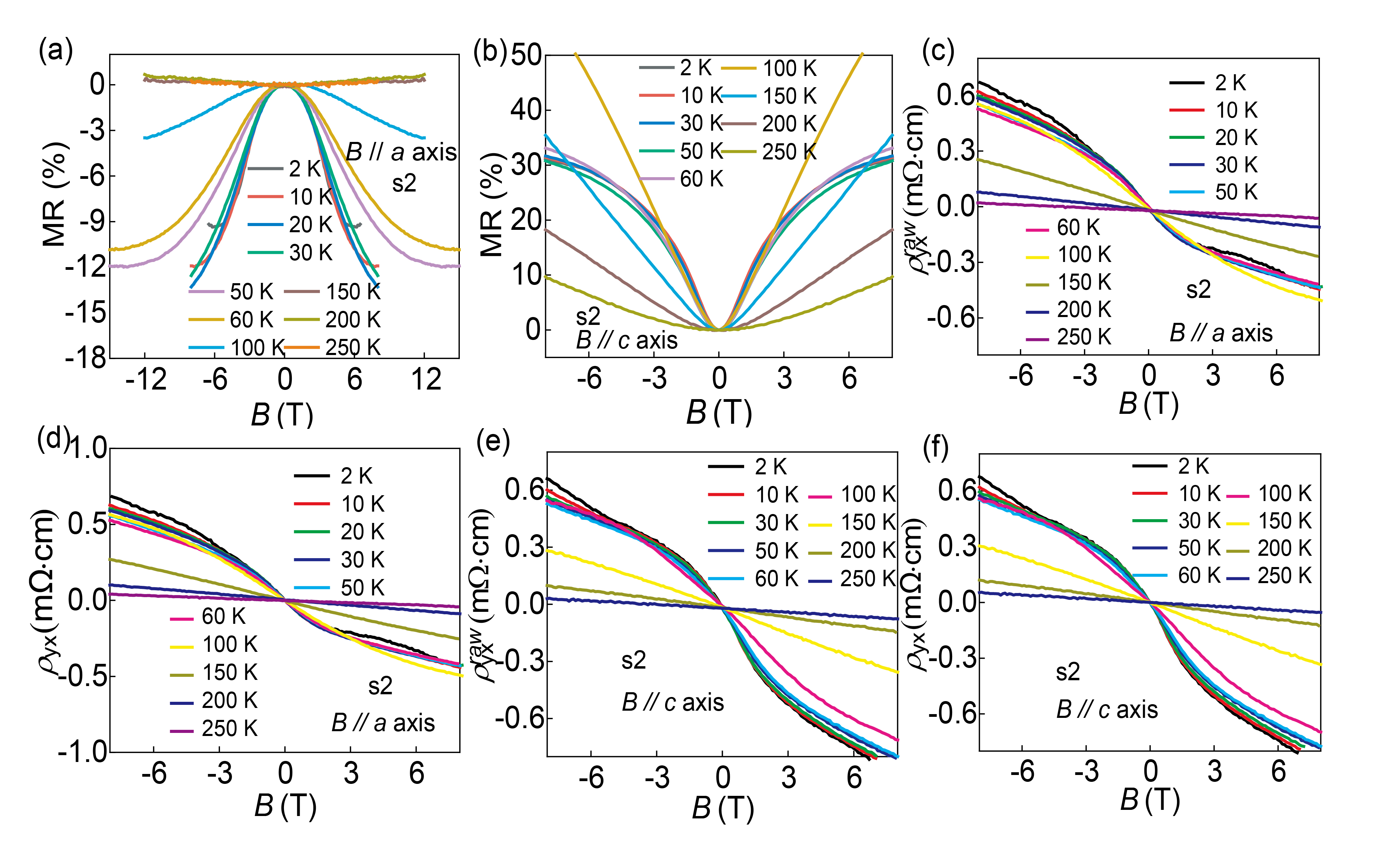


Fig. S2. Magnetoresistance ratio, Hall resistivity vs. B at $B\parallel I$($\theta=0^{\circ}$) and $B\perp I$($\theta=90^{\circ}$) at various temperatures of ZrTe_5_ device s2. (a),(b) Magnetoresistance ratio vs B at *B* // *a* axis and *B* // *c* axis at various temperatures. (c),(d) Raw Hall data and data after subtracting $\rho_{xx}$ caused by electrodes misalignment at $\boldsymbol{B\parallel I}$at various temperatures. (e),(f) Raw Hall data and data after subtracting $\rho_{xx}$ caused by electrodes mismatch at $\boldsymbol{B\perp I}$at various temperatures.


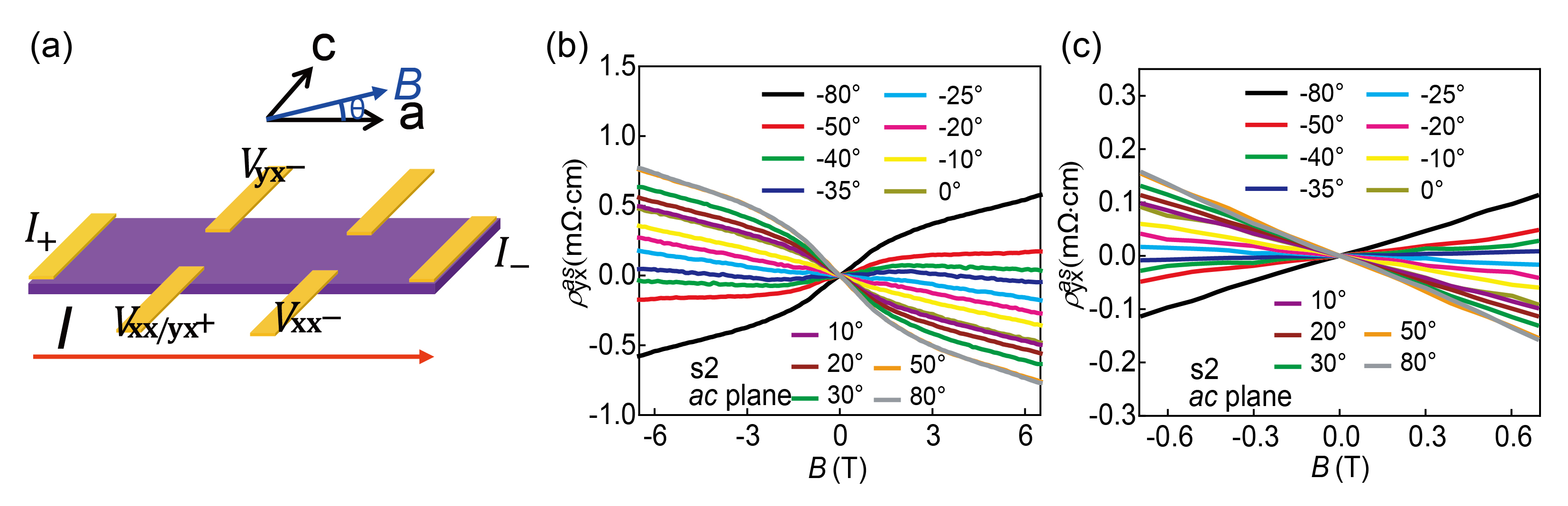


Fig. S3. Angular and temperature dependence of antisymmetric in-plane Hall resistivity of ZrTe_5_ device s2 detected in PPMS. (a) The schematic structure for the angular-dependent magnetotransport measurements in *ac* plane. (b) Hall resistivity detected at selected angles from -6.5 T to 6.5 T. (c) Hall resistivity detected at selected angles from -0.7 T to 0.7 T.


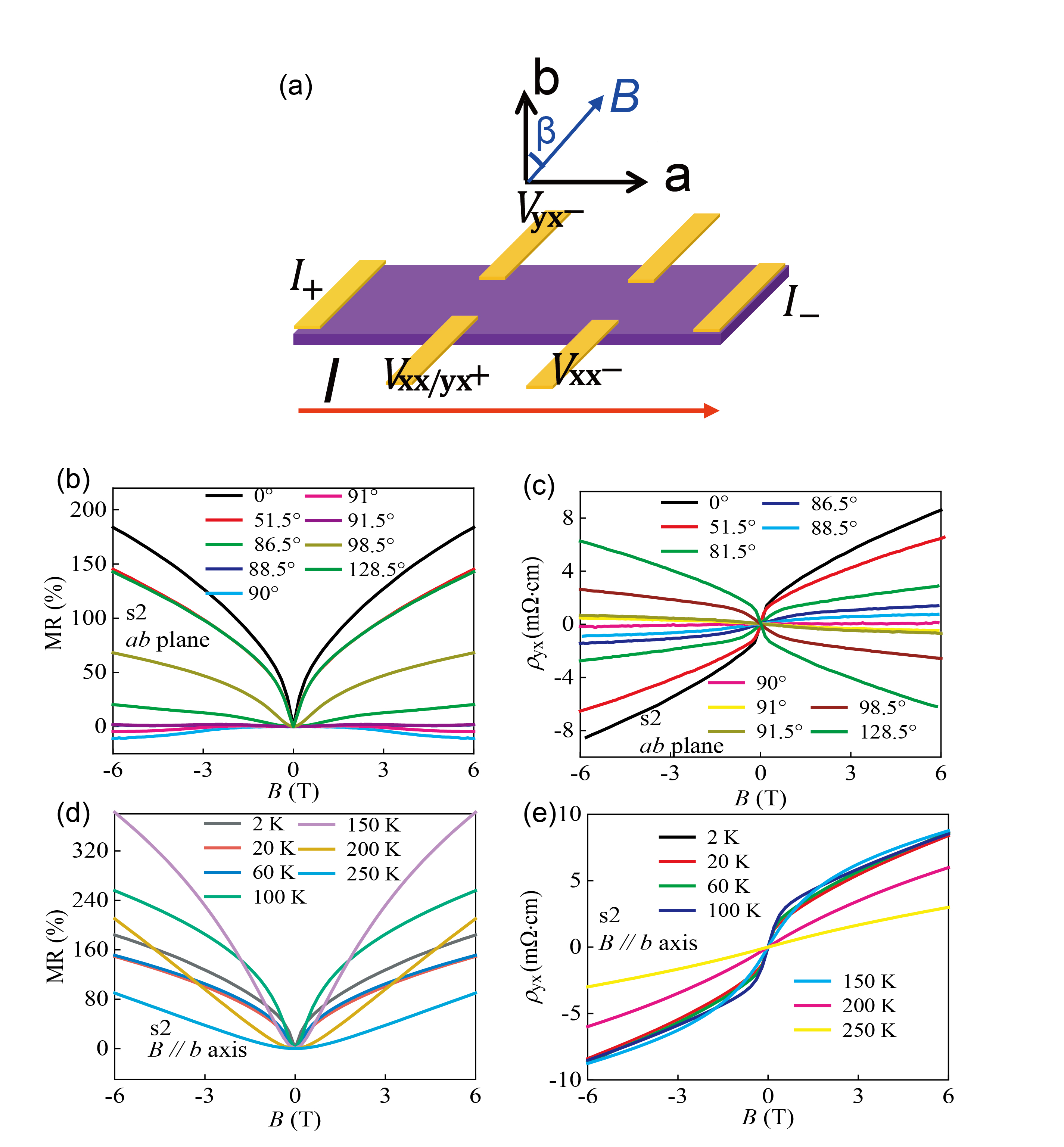


Fig. S4. Angular and temperature dependence of transport properties in *ab* plane of ZrTe_5_ device s2. Magnetic field lies in *ab* plane. (a) The schematic structure for the angular-dependent magnetotransport measurements in *ab* plane. (b),(c) MR behavior and Hall resistivity at selected angles. Negative LMR is detected in a range of 3$^{\circ}.$ (d),(e) MR and Hall resistivity versus *B* at various temperatures at $\boldsymbol{B\parallel}$*b* axis.


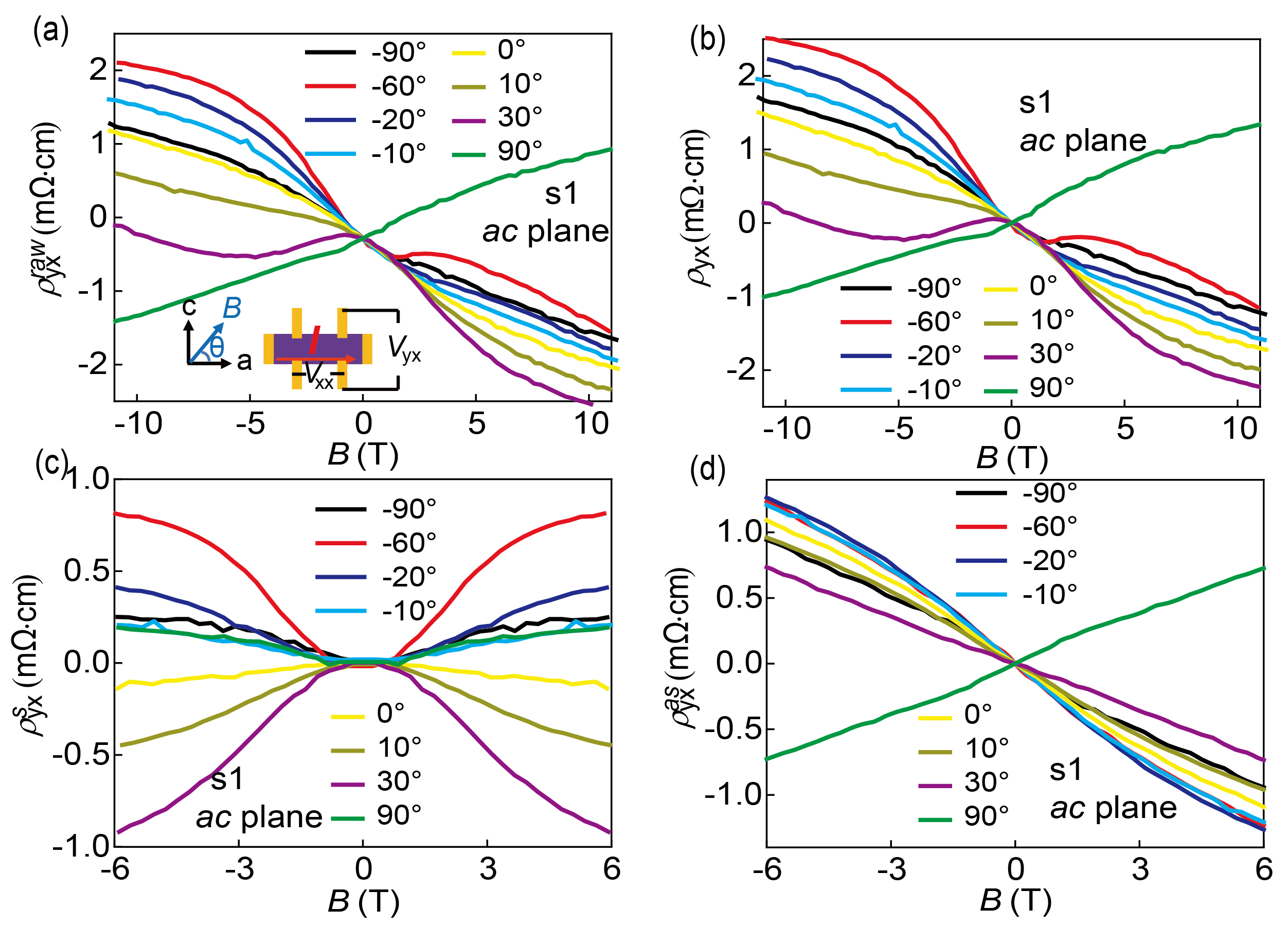


Fig. S5. Hall resistivity detected in ZrTe_5_ device s1. (a) Raw Hall resistivity versus *B* data at selected angles. Inset shows the schematic structure for the angular-dependent magnetotransport measurements in *ac* plane. (b) Hall resistivity at selected angles after subtracting $\rho_{xx}$ caused by electrodes mismatch. (c),(d) symmetric and antisymmetric in-plane Hall resistivity versus *B* at selected angles.

**References**

1. D. Xiao, M.-C. Chang & Q. Niu, Berry phase effects on electronic properties. Rev. Mod. Phys. **82**, 1959–2007 (2010).
2. M.-C. Chang & Q. Niu, Berry phase, hyperorbits, and the Hofstadter spectrum. *Phys. Rev.Lett.* **75**, 1348–1351 (1995).
3. M.-C. Chang & Q. Niu, Berry phase, hyperorbits, and the Hofstadter spectrum: Semiclassical dynamics in magnetic Bloch bands. *Phys. Rev. B* **53**, 7010–7023 (1996).
4. D. Xiao, J. Shi & Q. Niu, Berry Phase Correction to Electron Density of States in Solids. *Phys. Rev. Lett.* **95**, 137204 (2005).
5. H. Weng, X. Dai & Z. Fang, Transition-Metal Pentatelluride ZrTe_5_ and HfTe_5_: A Paradigm for Large-Gap Quantum Spin Hall Insulators. *Phys. Rev. X* **4**, 011002 (2014).
6. R. Y. Chen, Z. G. Chen, X.-Y. Song, J. A. Schneeloch, G. D. Gu, F. Wang & N. L. Wang, Magnetoinfrared Spectroscopy of Landau Levels and Zeeman Splitting of Three-Dimensional Massless Dirac Fermions in ZrTe_5_. *Phys. Rev. Lett.* **115**, 176404 (2015).
